# Isolation and Characterization of Human Dental Pulp Derived Stem Cells by Using Media Containing Low Human Serum Percentage as Clinical Grade Substitutes for Bovine Serum

**DOI:** 10.1371/journal.pone.0048945

**Published:** 2012-11-14

**Authors:** Federico Ferro, Renza Spelat, Antonio Paolo Beltrami, Daniela Cesselli, Francesco Curcio

**Affiliations:** 1 Department of Medical and Biological Sciences, University of Udine, Udine, Italy; 2 Regenerative Medicine Centre (CIME), Udine, Italy; National Institutes of Health, United States of America

## Abstract

Adult stem cells have been proposed as an alternative to embryonic stem cells to study multilineage differentiation *in vitro* and to use in therapy. Current culture media for isolation and expansion of adult stem cells require the use of large amounts of animal sera, but animal-derived culture reagents give rise to some questions due to the real possibility of infections and severe immune reactions. For these reasons a clinical grade substitute to animal sera is needed. We tested the isolation, proliferation, morphology, stemness related marker expression, and osteoblastic differentiation potential of Dental Pulp Stem Cells (DPSC) in a chemically defined medium containing a low percentage of human serum, 1.25%, in comparison to a medium containing 10% Fetal Bovine Serum (FBS). DPSCs cultured in presence of our isolation/proliferation medium added with low HS percentage were obtained without immune-selection methods and showed high uniformity in the expression of stem cell markers, proliferated at higher rate, and demonstrated comparable osteoblastic potential with respect to DPSCs cultured in 10% FBS. In this study we demonstrated that a chemically defined medium added with low HS percentage, derived from autologous and heterologous sources, could be a valid substitute to FBS-containing media and should be helpful for adult stem cells clinical application.

## Introduction

Transplantation of tissues and organs generated from allogeneic embryonic stem cells requires large manipulations and still carries many questions. Thus, although embryonic stem cell research provides a promising alternative solution to the problem of a limited supply of organs for transplantation, the problems and risks associated with the need for immunosuppression to sustain transplantation of allogeneic cells or tissue and questions on their safety, such as teratoma formation still remain [1]. Using cells from a post-natal individual, rather than an embryo, as a source of autologous or allogeneic stem cells would overcome the biological and clinical problems associated with the use of embryonic stem cells, as well as solve the ethical dilemma associated with embryonic stem cell research. A number of stem cells have been isolated from fully-developed organisms, particularly humans, but these cells culture protocols involve large use of animal sera [2], such as FBS, or horse serum and that is associated with many problems: the composition of animal serum is unknown and varies between batches, interfering with the reproducibility of experiments and they may be contaminated with viruses, mycoplasms, prions or other pathogenic, toxic or immunogenic agents [3]–[6]. Because of such safety risks, regulatory authorities discourage or prohibit the use of animal sera and other components for the production of biological products for human use [7]. For these reasons we developed and tested a chemically defined culture medium added with a small amount of autologous and heterologous human serum, which allowed us to isolate a highly proliferative population of dental pulp stem cells (DPSC), which expressed embryonic as well as mesenchymal stem cell markers and showed osteoblastic differentiation capacity comparable to a medium containing higher FBS amounts.

## Materials and Methods

### Isolation and culture of Dental Pulp Stem Cells

After written informed consent of donors' parents and ethics approval from the Ethics Committee of the Medical Faculty of Udine, dental pulps derived from normal exfoliated human deciduous teeth, of 5 to 9-year-old children (24 subjects), were extracted using a syringe needle and were transferred into 35-mm Petri dishes (Falcon, BD-Biosciences, San Jose, CA, USA). To test the best suitable HS percentage, capable of isolate and expand DPSCs, dental pulps were cultured in presence of an isolation/proliferation medium, as described by Ferro et al. [8], [9], [10], supplemented with 2.5%-1.25%-0.5%-0.25% human serum (HS). For comparison, dental pulps were also isolated and cultured in basal medium, composed of F-12 Coon's and Ambesi's modified (Gibco-Invitrogen, Carlsbad, CA), Medium-199 and CMRL-1066 (Sigma-Aldrich, St. Louis, MO, USA), added with growth factors alone or in basal medium supplemented with 1.25% human serum alone. Human serum was obtained after written informed consent of the donors. DPSCs were not subjected to any type of depletion techniques and when reached confluence were detached by trypsin (Sigma), and sub-cultured in 100 mm dishes at the density of 2×10^3^ cells/cm^2^. The culture was maintained semi-confluent in order to prevent the differentiation of the cells, and medium was changed every 3 days.

5×10^4^ DPSCs at passage 5 (P5), plated in triplicate, in 60 mm dishes, were used to generate growth curves in presence of media with or without different human serum percentages, as previously described, and were counted every day from day 1 to day 5, without medium changing.

DPSCs were also isolated and cultured in DMEM (Sigma) added with 10% FBS, 25 µg/ml gentamycin (Gibco) and in isolation/proliferation medium added with 1.25% heterologous human serum (C-HS), derived from commercially available human male AB plasma (Sigma).

To test and compare DPSCs proliferative capacity 5×10^4^ DPSCs at P5, isolated by using 1.25% HS, 10% FBS and 1.25% C-HS media, were plated in triplicate in 60 mm dishes and used for generate growth curves counting the cells at day 1, 3, 4, 5 with medium changing at day three. Human embryonic carcinoma stem cells (Ntera2), purchased from ATCC (ATCC-LGC, Milan, IT), were used as positive control for embryonic stem markers as suggested by Liedtke et al. [11], and were cultured according to Gallagher et al. [12]. Human osteoblast like cells, hOB, (ATCC-LGC, Milan, IT) were used as positive control for osteoblastic differentiation and were cultured by the method [13]. Human primary thyroid cells were cultured as already described by [14], and used as osteoblastic negative control. 1301 cell line, T-lymphoblastic leukemia, (Sigma) was maintained in RPMI 1640 medium (Gibco) supplemented with 10% FBS, 100 U/ml penicillin and 100 µg/ml streptomycin and was used as reference in flow-FISH analysis. Cells were counted in triplicate using a Neubauer chamber (Marienfeld GmbH, Lauda-Konigshofen, Ge) and 0.4% trypan blue (Sigma) solution was used to highlight non-viable cells. Population doubling times were calculated, in triplicate, during logarithmic growth phase by using doubling time software v1.0.10 (http://www.doubling-time.com) [15]. The number of total cell generations was obtained by dividing total culture time, expressed in hours and calculated from data obtained from P0 to P2, for the doubling time calculated from data obtained at P0 and P2, as previously reported. Cell doubling exponential rate and the number of total generations served us to evaluate the number of total cells starting from two progenitor cells. Then total cells, calculated from data obtained from P0 to P2, were divided by the number of total cells starting from two progenitor cells, estimating approximately the number of the primary culture progenitor cells. Media depletion time represents the time when the number of dead cells were approximately ≥10% with respect to the viable cells.

### Immunofluorescence (IF)

Immunofluorescence were performed on 4% paraformaldehyde fixed cells cultured in 1.25% HS, 1.25% C-HS and 10% FBS media at P5. Staining was performed using primary antibodies overnight at 4°C followed by incubation with conjugated secondary antibodies at room temperature for one hour. Primary antibodies were Nanog diluted 1∶125, Oct4 diluted 1∶125 SSEA-4 diluted 1∶50 (Abcam, Cambridge, MA), Sox-2 diluted 1∶200, TRA1–60 diluted 1∶40, TRA1–81 diluted 1∶40, SSEA-1 diluted 1∶50, SSEA-3 (Chemicon, Temecula, CA) diluted 1∶75, Secondary Antibodies were α-mouse IgM TRITC (Jackson, Sacramento, CA), α-mouse IgG FITC, α-Rabbit IgG FITC (Sigma), α-Goat IgG Alexa546 (Molecular Probes, Eugene, OR), α-Mouse IgM FITC, α-Rat IgG FITC (Abcam). Nuclear counter-staining was performed using DAPI (Pierce, Rockford, IL). Images were obtained using Leica DMI 6000B microscope connected to a Leica DFC350FX camera (Leica Microsystems).

### Fluorescence Activated Cell Sorting (FACS)

FACS analysis was performed on P5 cells cultured in 1.25% HS, 1.25% C-HS and 10%FBS media, after being detached from culture dishes. Staining was performed using properly conjugated primary antibodies (0.1 µg/10^6^ cells) CD-10, CD13, CD29, CD34, CD44, CD45, CD49, CD59, CD73, CD90, CD117 (All from BD, San Jose, CA), CD49a (BD-Pharmingen), CD105 (Serotec, Raleigh, NC), CD133 (Miltenyi Biotec, Bergisch Gladbach, GE). Conjugated isotype-matching antibodies were used as negative controls. Data (20.000 events) were collected from three independent experiments using a FACS-Calibur (BD) and expressed as mean ± standard deviations (SD).

### Flow-FISH

Telomeric sequences of DPSCs at P5, cultured in 1.25% HS, 1.25% C-HS and 10%FBS media, were evaluated by using flow-FISH telomere kit, (DakoCytomation, Glostrup, DK), by means of a FITC-conjugated peptide nucleic acid (PNA) probe, according to the manufacturer's instructions.

### Telomeric Repeat Amplification Protocol (TRAP)-assay

Pellets obtained from 1×10^6^ of P5 DPSCs cultured in 1.25% HS, 10% FBS and 1.25% C-HS media, were washed once with PBS, re-pelleted and resuspended in 200 µL of 1× lysis Buffer. The detection of telomerase reverse transcriptase (TRT) activity was performed utilizing the TRAPeze kit (Chemicon) following manufacturer instruction; in addition we also used Ntera2 cell extracts as supplemental positive control.

### Alkaline phosphatase (ALP) assay

DPSC cells at P5, cultured in 1.25% HS, 1.25% C-HS and 10%FBS media, were fixed at −20°C in 4% Paraformaldehyde in PBS for 1–2 minutes and washed in PBS for 10 minutes. Cells were then stained with the alkaline phosphatase substrate 5-bromo-4-chloro-3-indolyl phosphate and nitroblue tetrazolium (BCIP/NBT, Sigma) for 5 to 10 minutes and rinsed in H2O.

### Osteoblastic differentiation

DPSCs at P5 cultured in 1.25% HS, 1.25% C-HS and 10% FBS media were plated in 100 mm dishes at a density of 4×10^4^ cells/cm^2^ and osteo-induced for 3 weeks as described by Ferro et al. [8]–[10].

### Real time PCR analysis

Total RNA was extracted from P5 undifferentiated DPSCs cultured in 1.25% HS, 1.25% C-HS and 10% FBS media, and P5 osteo-induced DPSCs, after one and three weeks of differentiation, using TRIzol (Gibco-Invitrogen, Carlsbad, CA). Real Time PCR was conducted using SYBR green (Roche, Mannheim, Ge) on a 96-well-plate using Lightcycler480 (Roche). The total volume (20 µl) of each PCR reaction contained SYBR Green PCR Master Mix (Roche), 10 ng cDNA, and 0.4 µM of each of the forward and reverse primers. Real Time PCR (n = 3) was performed using the following primer sequences, PCR product sizes, annealing temperatures and gene bank accession numbers: alkaline phosphatase (Alp), 5′cctgccttactaactccttagtgc 5′cgttggtgttgagcttctga, 114 bp, 59°C, NM_000478.4; collagen type I (Coll-I) 5′gggattccctggacctaaag 5′ggaacacctcgctctcca, 63 bp, 59°C NM_000088.3; Osteocalcin (Osc) 5′tgagagccctcacactcctc 5′acctttgctggactctgcac, 98 bp, 59°C, NM_199173.4; Osteonectin (Osn) 5′ttccctgtacactggcagttc 5′aatgctccatggggatga, 109 bp, 59°C NM_003118.2; Osteopontin (Osp), 5′gagggcttggttgtcagc 5′caattctcatggtagtgagttttcc, 129 bp, 59°C, NM_000582.2; runt-related transcription factor 2 (RUNX2) transcript variant 2, 5′cagtgacaccatgtcagcaa 5′gctcacgtcgctcattttg, 104 bp, 59°C, NM_001015051.3; β-actin, 5′ccaaccgcgagaagatga 5′ccagaggcgtacagggatag, 97 bp, 59°C, NM_001101.3.

The transcript amount of each gene was normalized to β-actin. Relative fold change in expression was calculated using the ΔΔCT method (CT values<30) with respect to undifferentiated cells.

### Statistical analysis

Statistical analysis was performed by Student's t-test. Data from the experiments are expressed as mean ± standard deviation (SD) of three independent experiments.

## Results

### Identification of the best suitable HS percentage

**Figure 1:**
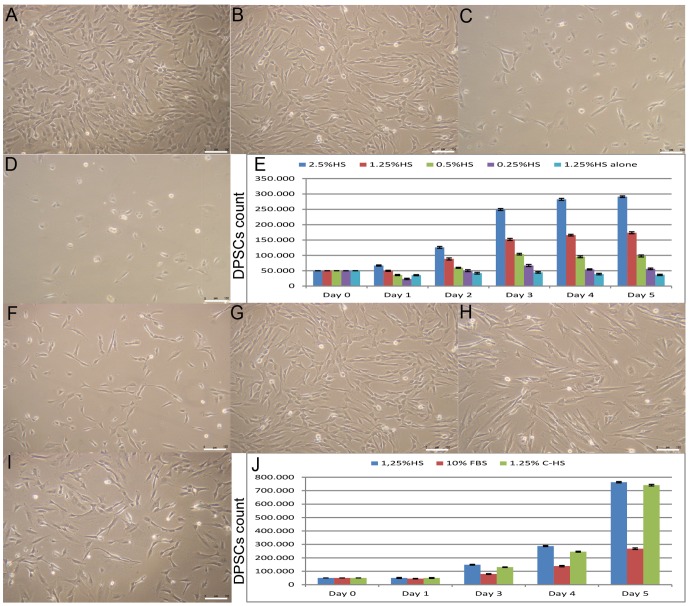
DPSC morphological characterization and growth curve. (A–F) Isolated DPSC were small, highly proliferative with reduced cytoplasm (A) in 2.5% HS medium, (B) in 1.25% HS medium, (C) in 0.5% HS medium, (D) in 0.25% HS medium. (E) 5×10^4^ DPSCs plated in 60 mm well, in presence of media added with 2.5% HS, 1.25% HS, 0.5% HS, 0.25% HS, and 1.25% HS medium alone, were maintained for 5 days without medium changing and used to generate growth curves. Estimated doubling time, expressed as mean ± SD, for 2.5–1.25% HS was 25.3±1.5 hours and 29.8±1.3 hours respectively. Doubling time for cells in media with 0.5%, 0.25% and 1.25% HS medium alone was 31.4±1.2, 31.4±2, and 146±1 hours respectively. X-DPSCs count Y-medium type, (p<0.05). (F) Morphology of DPSCs isolated and cultured in 1.25% HS medium alone. Bar scales 150 µm. (G–I) After two weeks DPSC isolated in 1.25% HS medium (G) displayed a homogeneous morphology with reduced cytoplasm and were small, highly proliferative with respect to DPSCs cultured in 10% FBS medium (H) and in 1.25% C-HS (I). Bar scales 150 µm. (J) 5×10^4^ DPSCs plated in 60 mm well were maintained for 5 days in culture, with medium changing at day three, and used to generate growth curves. Estimated doubling time, expressed as mean ± SD, was 28±2 hours in 1.25% HS, instead was 45±2.5 hours in presence of 10% FBS and 31.5±2 hours in 1.25% C-HS. X-DPSCs count Y-medium type, (p<0.05).

In order to identify the lowest HS percentage capable, in conjunction with our medium, to permit the best suitable isolation and culture condition for DPSCs, we cultured dental pulps, extracted using a syringe needle, in presence of different HS percentages 2.5-1.25-0.5-0.25%. Obtained DPSCs were not subjected to any type of common selection techniques (immuno-depletion, physical centrifugation or filtration and chemical depletion by erythrolysis); also we did not use murine feeder layers, fibronectin or other adhesion protein layers. From two to five weeks after plating, depending on HS percentages, DPSC were small, highly proliferative and exhibit a homogeneous, fibroblastoid morphology with scanty cytoplasm (Fig. 1A–D).

To evaluate media efficiency we used two indicators: 1- population doubling time, 2- medium depletion time. Growth curves confirmed that DPSC proliferate at high rate both in media added with 1.25–2.5% HS with respect to 0.25–0.5% HS (level of significance p<0.05). We noted a gradual decreasing in plating efficiency with lowering serum and cell population doubling time of DPSCs growing in medium containing 1.25–2.5% HS was about 25.3±1.5 hours and 29.8±1.3 hours respectively (Fig. 1E and Table 1) (p<0.05), while that of cells growing in medium containing in 0.5–0.25% HS and was 31.4±1.2, 31.4±2 hours respectively (Fig. 1E and Table 1). Medium depletion time in tested conditions 2.5% HS, 1.25% HS, 0.5% HS and 0.25% HS was respectively 5days, 4days, 3days and 2days. On the contrary, DPSCs isolated and cultured in basal medium (F-12 Coon's and Ambesi's modified, Medium-199 and CMRL-1066) added with 1.25% HS alone evidenced a similar morphology (Fig. 1F), but population doubling time and medium depletion time were 146±1 hours and 2days respectively (Fig. 1E and Table 1). Instead we found that dental pulps cultured in isolation/proliferation medium without HS did not develop colonies and did not proliferate. All together these data allowed us to identify the medium added with 1.25% HS as the best suitable alternative to media containing lower serum percentages, due to its capacity to permit higher proliferation rate, as well as its higher medium depletion time. For these reasons, and because our intentions were to save and reduce as much as possible HS percentage, we decided to use isolation/proliferation medium added with 1.25% HS in the following comparison experiments, even with respect to media containing higher HS percentage.

**Table 1:** Testing diverse HS percentages.

Medium	Day 0	Day 1	Day 2	Day 3	Day 4	Day 5
**2.5%HS**	50.000±100	67.000±1.912	126.038±3.001	249.272±3.067	282.352±3.234	291.012±2.456
**1.25%HS**	50.000±100	49.800±2.834	88.392±3.304	151.982±3.545	165.847±2.756	173.737±3.134
**0.5%HS**	50.000±100	36.082±1.789	59.629±1.678	104.007±2.679	95.205±2.908	98.162±3.268
**0.25%HS**	50.000±100	23.261±2.234	50.312±3.056	67.100±3.456	54.353±1.904	56.099±2.345
**1.25%HS alone**	50.000±100	35.590±1.789	42.008±2.912	44.600±2.567	39.087±2.405	36.660±1.989

### HS and FBS media comparison

Consequently, we compared our isolation/proliferation medium capabilities with a commercial medium added with 10% FBS. In addition, we also tested and compared the properties of our medium substituting HS with 1.25% of a commercial human serum (C-HS) which can be more easily accessible, facilitating the establishment of a large scale up production process. Morphologically cells cultured in 1.25% HS (Figure 1G) were more homogenous with fibroblastic shape with respect to cells cultured in 10% FBS (Figure 1H) and in 1.25% C-HS (I). Growth curves confirmed that DPSC proliferate at a similar rate in our medium added with 1.25% HS, 28±2 hours, as well as in medium added with 1.25% C-HS, 31.5±2 hours, and both proliferate at higher rate with respect to 10% FBS medium, 45±2.5 hours (Fig. 1J and Table 2) (p<0.05). The estimated number of stem progenitor cells in all primary culture conditions varied from 80 to 800. Moreover we evidenced that in both culture conditions the presence of a low HS percentage permitted to DPSC to be detached more quickly with respect to the same cells cultured in 10% FBS, approximately 25% lesser, without compromising cell adhesion.

**Table 2:** HS and FBS comparison.

Medium	Day 0	Day 1	Day 3	Day 4	Day 5
**1.25%HS**	50.000±100	50.200±3.765	148.250±2.901	287.375±4.012	763.125±5.086
**10% FBS**	50.000±100	45.000±1.997	79.375±3.045	138.250±5.051	268.125±5.890
**1.25% C-HS**	50.000±100	49.800±3.405	130.620±2.087	245.690±3.154	741.245±5.604

### Cluster differentiation markers expression

Additionally, the cells outgrown from the seeded fragments cultured in 1.25% HS and 1.25% C-HS medium were uniformly positive for human mesenchymal stem cell surface antigens (CD)(Table 3), expressing high levels (≥92±5%) of CD10, CD29, CD44, CD49a, CD49d, CD59, CD73, CD90, CD105 and low levels of CD133 2±0.1%, CD117 15±2% [8], [16]. Moreover were less positive for CD34 1%±0.1 and CD45 1%±0.1 ruling out contamination with hematopoietic differentiated cells, although they show a strong positivity for CD13 95±5 [8], [16] (Table 3) (p<0.05). Instead, primary DPSCs cultured in 10% FBS showed a lower uniformity for what about concerns CD10, CD13, CD29, CD44, CD49a, CD49d, CD117 and CD133, instead CD34, CD45, CD59, CD73, CD90, CD105 percentages were similar to that obtained for DPSC cultured in 1.25% HS and 1.25% C-HS media (Table 3) (p<0.05). To confirm 1.25% HS medium properties, DPSCs cultured in 1.25% HS medium were splitted into the 10% FBS commercial medium and tested by FACS for the same CDs, results demonstrated a slightly decreased expression for CD44, CD90, CD105 and an increase in CD10, CD13 with respect to data obtained in 1.25% HS (Table 3) (p<0.05).

**Table 3:** Cluster differentiation markers expression.

Marker	1.25% HS	10% FBS	Splitted	1.25% C-HS
CD10	92±5%	15±3%	95±4%	93±4%
CD13	95±5%	45±5%	99±1%	92±6%
CD29	98±2%	87±4%	98±1%	95±5%
CD34	1±0.1%	0.1±0.01%	2.5±0.8%	1±0.2%
CD44	99±1%	76±8%	89±4%	99±1%
CD45	1±0.1%	0.27±02%	0.15±1%	1±0.3%
CD49a	97±3%	87±5%	98±2%	98±1%
CD49d	99±1%	63±10%	98±1%	98±2%
CD59	99±1%	99±1%	99±1%	99±1%
CD73	99±1%	99±1%	99±1%	99±1%
CD90	99±1%	97±3%	84±3%	99±1%
CD105	97±3%	97±6%	75±5%	97±2%
CD117	15±2%	1±0.1%	2.3±1%	10±5%
CD133	2±0.1%	0.5±0.5%	0.57±0.9%	1±0,3%

### Embryonic stem markers expression

**Figure 2:**
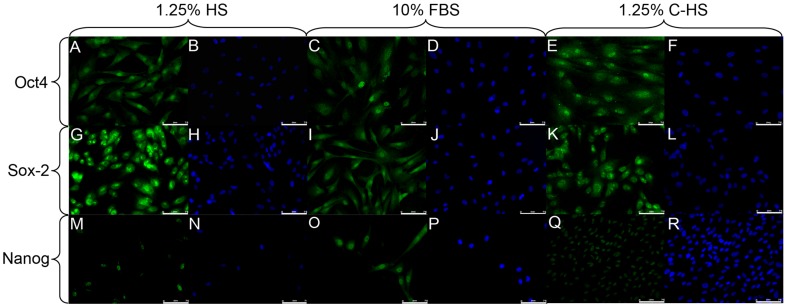
IF analysis for ES transcription factors. (A–F): FITC labeled antibody was used to evaluate, by IF, Oct4 protein expression in DPSCs cultured in 1.25% HS (A, B), 10% FBS (C, D) and 1.25% C-HS (E, F). (G–L): FITC labeled antibody was used to evaluate, by IF, Sox-2 expression in DPSCs in proliferation in 1.25% HS (G, H) 10% FBS (I, J) and 1.25% C-HS (K, L). (M–R): FITC labeled antibody was used to evaluate, by IF, Nanog expression for DPSCs in proliferation in 1.25% HS (M, N) 10% FBS (O, P) and 1.25% C-HS (Q, R). Bar scales 75 µm.

**Figure 3:**
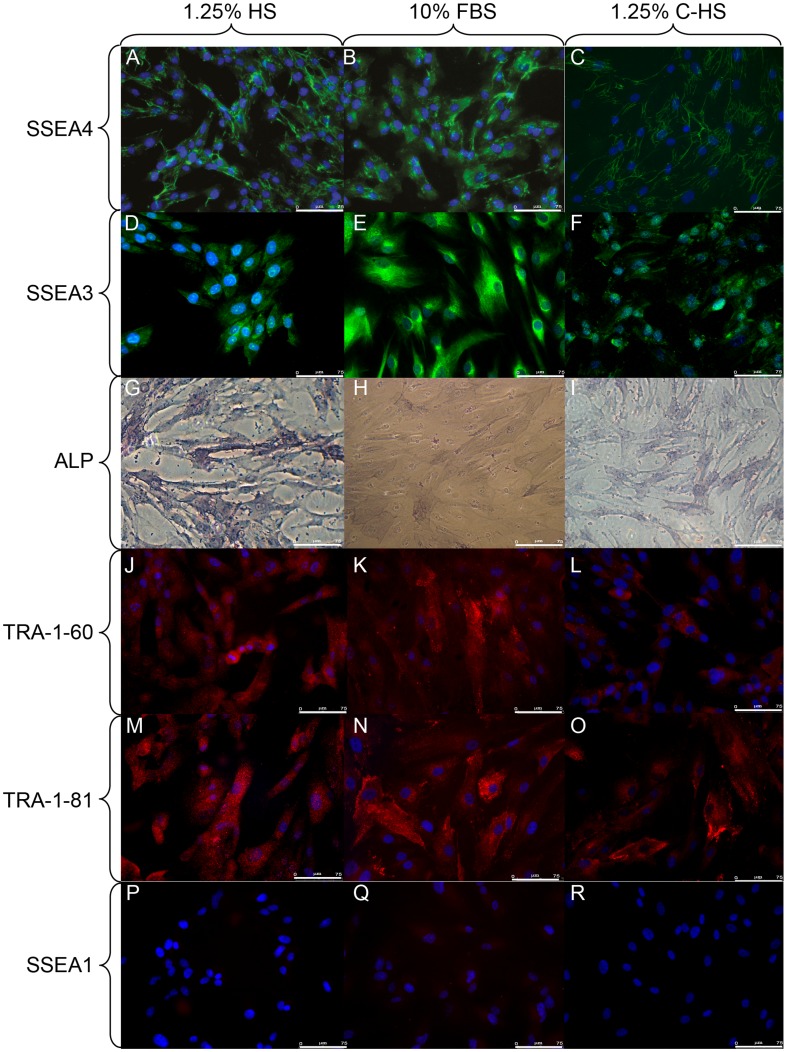
IF for ES membrane markers. FITC labeled antibody was used to evaluate, by IF, SSEA4 expression in DPSCs cultured in 1.25% HS (A), 10% FBS (B) and 1.25% C-HS (C) in expansion condition. DPSCs were reacted with FITC labelled anti-SSEA3 antibody evidencing its expression in 1.25% HS (D), 10% FBS (E) and 1.25% C-HS (F) media. Alkaline Phosphatase assay demonstrates higher enzymatic activity in DPSC cultured in 1.25% HS (G) with respect to DPSCs cultured in 10% FBS (H) and 1.25% C-HS (I). TRITC labeled antibodies were used to evaluate, by IF, TRA-1–60 expression in DPSCs cultured in 1.25% HS (J), 10% FBS (K), 1.25% C-HS (L) and TRA-1–81 in DPSCs cultured in 1.25% HS (M), 10% FBS (N) and 1.25% C-HS (O). SSEA-1 immuno-reactivity was not evident in both culture conditions 1.25% HS (P), 10% FBS (Q) and 1.25% C-HS (R), using TRITC labeled antibody. Bar scales 75 µm.

Immunofluorescence shows that in DPSC Oct4 is localized not only in nuclei, but also in cytoplasm in both culture conditions, respectively 1.25% HS, 10% FBS, and 1.25% C-HS [8] (Fig. 2A, B; 2C, D; 2E, F), as well as Sox-2 (Fig. 2G, H; 2I, J; 2K, L). Nanog had nuclear expression in DPSCs cultured in media added with 1.25% HS and 1.25% C-HS, instead showed both nuclear and cytoplasmic expression in 10% FBS medium (Fig. 2M, N; 2O, P; 2Q, R). The high fraction of cells expressing Oct4, Sox-2 and Nanog denote that DPSCs are an uniform population of stem cells [16], [17], as indicated also by the expression of the globoseries glycolipid antigens SSEA-4 (Fig. 3A, 3B, 3C), SSEA-3 [16], [18] (Fig. 3D, 3E, 3F) and the ALP positivity [19], evidenced by colorimetric assay, (Fig. 3G, 3H, 3I) respectively in 1.25% HS, 10% FBS and 1.25% C-HS media. Other undifferentiated embryonic stem (ES) markers the keratan sulphate-relate antigens TRA-1–60 (Fig. 3J, 3K, 3L), TRA-1–81 (Fig. 3M, 3N, 3O) were uniformly expressed in DPSCs; while SSEA-1, (Fig. 3P, 3Q, 3R) the lactoseries oligosaccharide antigen, expressed in differentiated ES cells, was not evidenced [16], [20].

### Telomere and TRT activity assays

**Figure 4:**
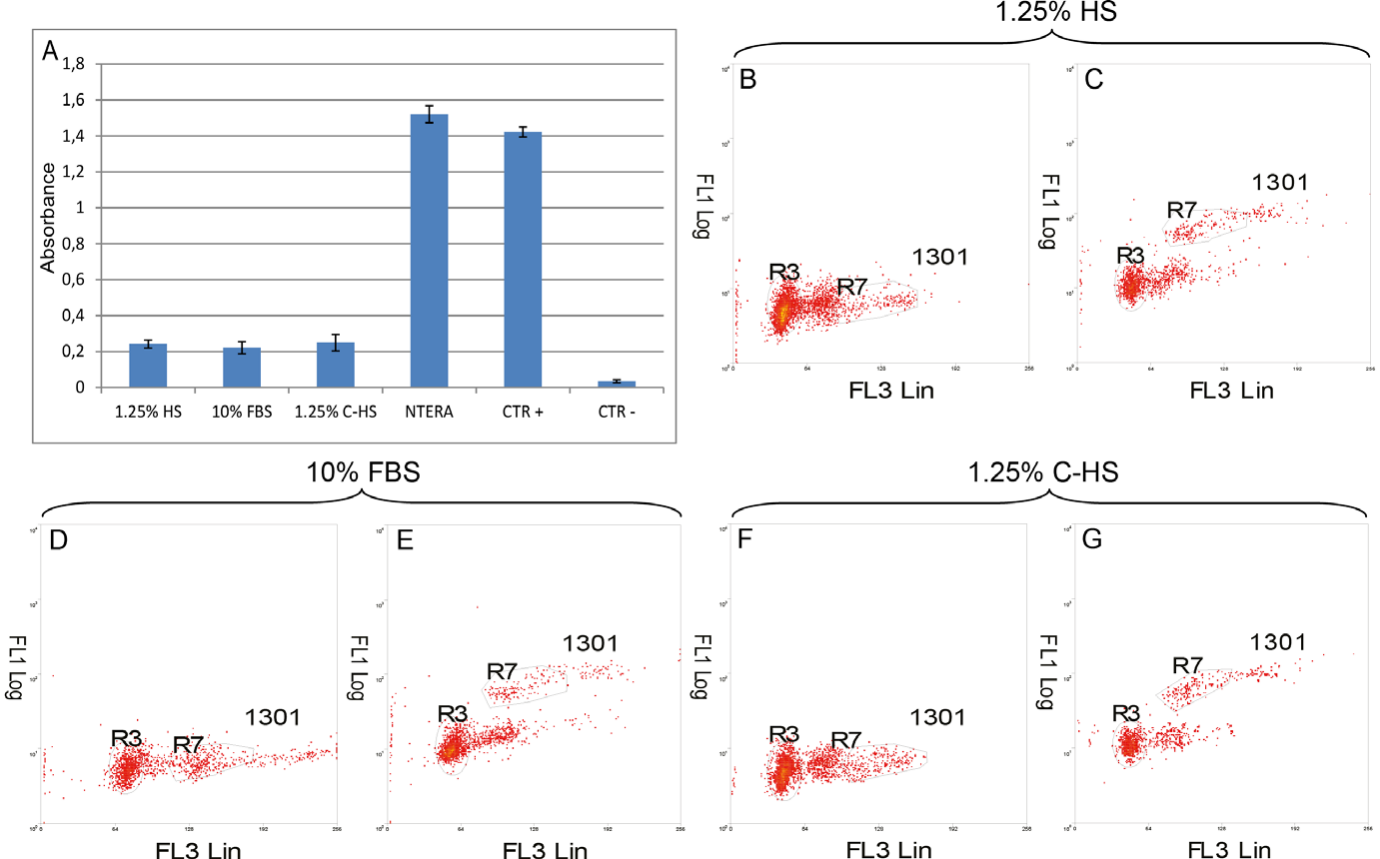
Telomerase and telomeres. (A) Graph evidence the relative TRT activity of DPSCs cultured in 1.25% HS medium, 10% FBS and 1.25% C-HS media compared to Ntera2 and positive control (Ctr +). X-absorbance Y-cell types, (p<0.05). (B–G) Dot plot of FL1-height versus FL3-height of cells hybridized with hybridization solution without Telomere PNA Probe. Gates are set around cells in the G0/1- phase for both sample cells, DPSC in 1.25% HS medium (R7) (B), DPSC in 10% FBS (R7) (D) DPSC in 1.25% C-HS medium (R7) (F) and control cells (1301 cell line). Dot plot of FL1-height versus FL3-height of cells hybridized with Telomere PNA Probe/FITC in Hybridization Solution. Gates are set around cells in the G0/1-phase for both sample cells DPSC in 1.25% HS medium (R7) (C), DPSC in 10% FBS (R7) (E) DPSC in 1.25% C-HS medium (R7) (G) and control cells (1301 cell line). The above relative telomere length (RTL) value indicates that the average telomere fluorescence per chromosome/genome in DPSCs in 1.25% HS medium, 10% FBS and 1.25% C-HS was about 18±1.1%, 17±0.9% and 17.1±1.1% respectively of the telomere fluorescence per chromosome/genome in the control cells (1301 cell line) (p<0.05).

Several reports have demonstrated the role of telomere length and telomerase activity in stem cell self-renewing, ageing and mobilization processes [21], [22]. In order to verify whether cells growing under our experimental conditions possessed telomerase activity, a TRAP assay was performed observing that cells displayed in both culture conditions a low but present TRT activity 15±0.7% in 1.25% HS, 14±0.8% in 10% FBS, and 14.8±0.5% in 1.25% C-HS with respect to Ntera2 cells, and 17±0.5% in 1.25% HS, 15±0.3% in 10% FBS and 17±0.8% in 1.25% C-HS with respect to manufacturer positive control (Fig. 4A and Table 4) (p<0.05); Telomere length of DPSCs, as assessed by Flow-FISH analysis, was, relatively to the telomeric length of the 1301 cell line, and was18±1.1% (R7) (Fig. 4C) in 1.25% HS, 17±0.9% (R7)(Fig. 4E) in 10% FBS and 17.1±1.1% (R7) (Fig. 4G) in 1.25% C-HS respectively. For comparison, the average telomeric length of cord blood cells was 18.5±3.9%, when compared to 1301 cell line [23] (p<0.05).

**Table 4:** Telomerase reverse transcriptase activity.

Samples	TRT activity	TRT % Ntera2	TRT % Ctr +
**1.25% HS**	0.242±0.023	15±0.7%	17±0.5%
**10% FBS**	0.221±0.034	14±0.8%	15±0.3%
**1.25% C-HS**	0.250±0.045	14.8±0.5%	17±0.8%
**NTERA**	1.521±0.048	1	
**CTR +**	1.422±0.028		1
**CTR −**	0.035±0.009		

### Osteogenic induction

**Figure 5:**
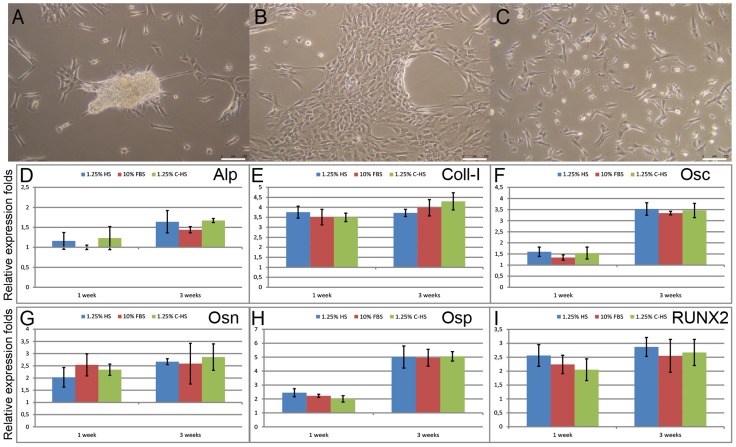
Testing and comparing osteoblastic differentiation capacity. (A–C) DPSCs cultured in 1.25% HS (A), 10% FBS (B) and 1.25% C-HS (C) were osteo-induced for three weeks, and during this period DPSCs changed their morphology, developing an asymmetric shape with an enlarged end. Bar scales 150 µm. (D–I) Real Time PCR showed a comparable increased expression for all the tested markers with no significant differences deriving from the different proliferation conditions, both after one and three weeks of differentiation: 1.25% HS (blue), 10% FBS (red), 1.25% C-HS (green). X- relative expression folds Y- osteo-induction days.

In order to compare differentiation properties, proliferating DPSCs cultured in 1.25% HS, 10% FBS and 1.25% C-HS media were osteo-induced for three weeks. During the osteoblastic inductive period cells changed their fibroblastoid morphology, developing an asymmetric shape with an enlarged end (Fig. 5A, B, C), as previously demonstrated by Ferro et al. [8]

In addition, the osteo-specific genes expression pattern for alkaline phosphatase (Alp) (D), collagen type I (Coll-I) (E), osteocalcin (Osc) (F), osteonectin (Osn) (G), osteopontin (Osp) (H), and runt-related transcription factor 2 (RUNX2) transcript variant 2 (I) were analyzed at mRNA level both after one and three weeks of osteo-induction.

Real Time PCR data, expressed as differentiated over undifferentiated DPSC, showed an increased and comparable expression, both at one and three weeks of osteoblastic induction, for all the tested markers with no significant differences deriving from the different proliferating conditions, as evidenced in Table 5 and Figure 5D–H.

**Table 5:** Osteoblastic differentiation.

Alp	1 week	2 weeks	Coll-I	1 week	2 weeks
**1.25% HS**	1.16±0.21	1.64±0.28	1.25% HS	3.76±0.3	3.72±0.18
**10% FBS**	1±0.054	1.44±0.078	10% FBS	3.51±0.39	3.98±0.41
**1.25% C-HS**	1.23±0.29	1.67±0.051	1.25% C-HS	3.5±0.21	4.3±0.43

Osc	1 week	2 weeks	Osn	1 week	2 weeks
**1.25% HS**	1.6±0.21	3.53±0.28	1.25% HS	2.03±0.4	2.67±0.12
**10% FBS**	1.34±0.12	3.34±0.078	10% FBS	2.54±0.45	2.59±0.83
**1.25% C-HS**	1.54±0.27	3.46±0.32	1.25% C-HS	2.34±0.23	2.86±0.54

Osp	1 week	2 weeks	RUNX2	1 week	2 weeks
**1.25% HS**	2.45±0.29	5.01±0.79	**1.25% HS**	2.56±0.39	2.87±0.34
**10% FBS**	2.23±0.11	4.96±0.6	**10% FBS**	2.24±0.33	2.55±0.59
**1.25% C-HS**	2.01±0.23	5.06±0.34	**1.25% C-HS**	2.05±0.39	2.67±0.47

## Discussion

Since early 20th century scientists have been searching for methods to allow the isolation and growth of tissues and cells outside of the body. In the late 1940's the first cell line (HeLa) was cultivated *in vitro* in a fluid mixture of chicken plasma, bovine embryo extract and serum from umbilical cord blood [24]. This crude mixture was the forerunner of today's modern cell culture media. The ideal culture medium simulates the exact *in vivo* conditions. In practice this can only be achieved to a limited extent as the physiological conditions required to cultivate tissues, cells and living organisms are extremely complex.

Serum provides all of the growth factors, vitamins, co-factors, hormones, attachment factors (fibronectin, laminin), transport factors (albumin, globulin, transferrin), nutrients (nucleosides, amino acids, fatty acids, lipids), trace elements and other factors which limit free radicals, toxins and heavy metals. Serum is a very complex product and only a small percentage of the components have been fully identified. For this reason, and in the absence of a valid alternative, it remains the most effective growth product for cell culture available today.

Most sera used in cell culture are from animal, mainly bovine origin [2]. This brings some disadvantages such as antibodies which may impair or damage cell growth, the possibility of presence of adventitious animal viruses and the possible contamination with endotoxins and mycoplasmas which can damage fragile cell lines.

With respect to the pathogenic risks due to addition of FBS in culture media, autologous HS is considered a safer alternative excluding the transfer of animal derived infections and related immunogenic reactions.

Therefore, to safely produce DPSCs for clinical applications, we formulated and tested an isolation/proliferation media, reducing as much as possible serum percentage presence and substituting it by adding specific cytokines and growth factors in order to obtain a well-defined composition.

It is likely that DPSC represent a small subpopulation of dental pulp resident cells that, under experimental conditions, were predominantly and selectively proliferating [8], [16], [25], [26]; moreover it has been reported that chemotactic gradient between the dental pulp fragment and the culture medium served as a vector directing the cells toward what is perceived as a site of injury, leading to their continuous and selective migration to the Petri dish [16]. For these reasons our approach has been developed in order to simplify the isolation procedure and overall to test the effective media capacity to isolate stem cells, starting from a source which contains a low number of stem progenitors as dental pulps. The high expression of markers found in embryonic stem cells, adult stem cells, the high proliferation rate, the TRT activity as well as the relatively long telomere presence, evidence that DPSCs have an expression profile that partially overlaps with either ES and adult stem cells and confirms their undifferentiated state [17], [27]. In addition our data demonstrate that osteoblastic differentiation potential is not negatively affected by our culture conditions, as evidenced by the Real Time PCR data and as previously demonstrated by us using the same methods and culture medium [8]. Such high proliferation rate, phenotype and differentiation capacity are consistent with results obtained using high 10–20% FBS percentages [16], [25], [26], demonstrating that a population of adult stem cells derived from human dental pulps could be obtained using a chemically defined medium which contains low HS percentage. More specifically, data evidenced that the coordinated action of the growth factors and the low HS percentage are responsible for this growth rate, because DPSCs cultured in presence of 1.25% HS alone or growth factors alone proliferate at lower rate or did not proliferate. The likeness in population purity between Immature Dental Pulp Stem cells (IDPSC) [16] and the population of cells described in the present study leads us to believe that they are similar. The difference consists in the use of a medium with very low human serum, derived from autologous and heterologous sources, which makes our medium more suited for human clinical applications. In addition medium capabilities were also confirmed starting from different adult stem cell sources, specifically from adipose tissue [9] and bone marrow [10]. In conclusion this medium hold strong promise in clinical reparative medicine for the treatment of degenerative or inherited diseases and are free of the ethical concerns raised by the use of ES. Moreover these data confirm that, in vision of a robust scale up process, even a commercial human serum, which can be more easily accessible, could be used to obtain similar results.

Autologous *ex vivo* expanded adult stem cells could be used for implantation aimed to repair damaged, aged or diseased tissues and organs. Finally the ability to stably transduce DPSC cells with specific genes would also enable the genetic manipulation of DPSC autologous cells for the treatment of degenerative and congenital disorders [28].
